# Resolution of Erythema Annulare Centrifugum With Lung Cancer Drug Therapy: A Case Report

**DOI:** 10.7759/cureus.73977

**Published:** 2024-11-19

**Authors:** Miwa Kagabu, Hidesato Odaka, Yuri Takita, Takuo Tokairin, Takuya Nagai

**Affiliations:** 1 Post Graduate Clinical Education Center, Japanese Red Cross Akita Hospital, Akita, JPN; 2 Department of Respiratory Medicine, Japanese Red Cross Akita Hospital, Akita, JPN; 3 Department of Pathology, Japanese Red Cross Akita Hospital, Akita, JPN; 4 Department of Dermatology, Japanese Red Cross Akita Hospital, Akita, JPN

**Keywords:** drug therapy, erythema annulare centrifugum, lung adenocarcinoma, lung cancer, osimertinib, paraneoplastic erythema annulare centrifugum eruption (peace), paraneoplastic skin syndrome

## Abstract

Tumor-associated erythema annulare centrifugum is commonly observed in leukemia and lymphoma. In this case, we reported erythema annulare centrifugum associated with lung cancer that resolved following targeted drug therapy. A 71-year-old man with psoriasis visited our hospital with erythema annulare and pruritus. Initially, he was prescribed cyclosporine at a dose of 2.5 mg/kg suspecting a worsening of his psoriasis; however, this treatment was ineffective. Signs of paraneoplastic skin syndrome were observed, leading to a diagnosis of epidermal growth factor receptor mutation-positive lung adenocarcinoma (cT3N2M1c, stage IV, exon 19 del). After initiating osimertinib at a dose of 80 mg, the skin rash improved, and the tumor size was reduced. The final diagnosis was paraneoplastic erythema annulare centrifugum associated with lung cancer. This case highlights the potential resolution of erythema annulare centrifugum through lung cancer therapy, emphasizing the importance of considering lung cancer differential diagnosis when this skin condition is present.

## Introduction

The number of lung cancer cases has increased, leading to more opportunities to treat patients with paraneoplastic syndrome. However, paraneoplastic skin syndromes remain rare in lung cancer [1-2]. Paraneoplastic skin syndromes involve hair growth disorders, musculoskeletal disorders, papulosquamous disorders, reactive erythemas, and vascular conditions [3]. Erythema annular centrifugum (EAC) is classified as a reactive erythema, and it presents with annular erythematous lesions resembling urticaria-like papules that enlarge centrifugally while clearing at the center [3-4]. EAC has been linked to underlying malignancies (paraneoplastic erythema annulare centrifugum eruption (PEACE)) such as leukemia and lymphoma [3,5]. The strategy for treating PEACE focuses on addressing the fundamental neoplastic process [3]. Osimertinib is an orally administered epidermal growth factor receptor tyrosine kinase inhibitor that specifically targets activating mutations of the epidermal growth factor receptor [6]. This precision therapeutic approach is designed to enhance treatment efficacy for patients with certain types of non-small-cell lung cancer. The effects of lung cancer drug therapy on this condition are not well understood. This report discusses a case in which EAC resolved as the lung cancer shrank due to drug treatment.

## Case presentation

**Figure 1:**
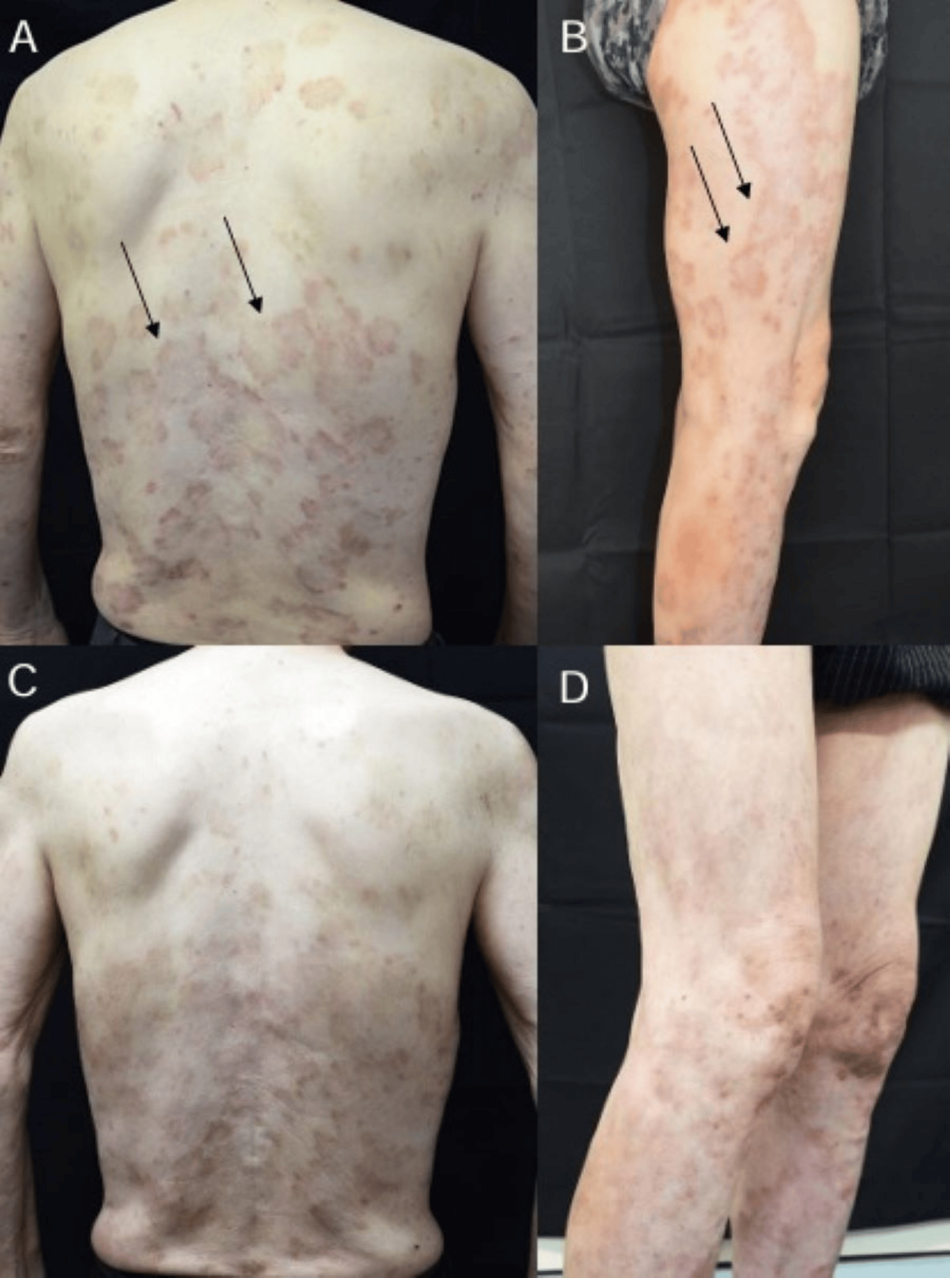
Erythema annulare changes. Erythema is characterized by concentric red rings that can be observed on the trunk (A) and extremities (B). These rings are single-layered and expand outward. The edges of the rings are slightly raised, exhibiting no signs of infiltration or desquamation (as indicated by the arrow). In some areas, the rings exhibit a C-shaped appearance. After one month of treatment with osimertinib, the erythema resolves, leaving only pigmentation (C, D).

A 71-year-old man was diagnosed with psoriasis at the age of 63 (eight years prior). He had been prescribed calcipotriol hydrate and betamethasone dipropionate for the condition. He previously smoked five cigarettes per day for 40 years from the age of 20 to 60. At age 66, he underwent surgery for ascending colon cancer. He reported no known allergies. He visited our hospital with frequent red rashes on his trunk and limbs, accompanied by worsening pruritus. Erythema characterized by concentric red rings was observed on the trunk and extremities (Figures 1A, 1B).

**Figure 2:**
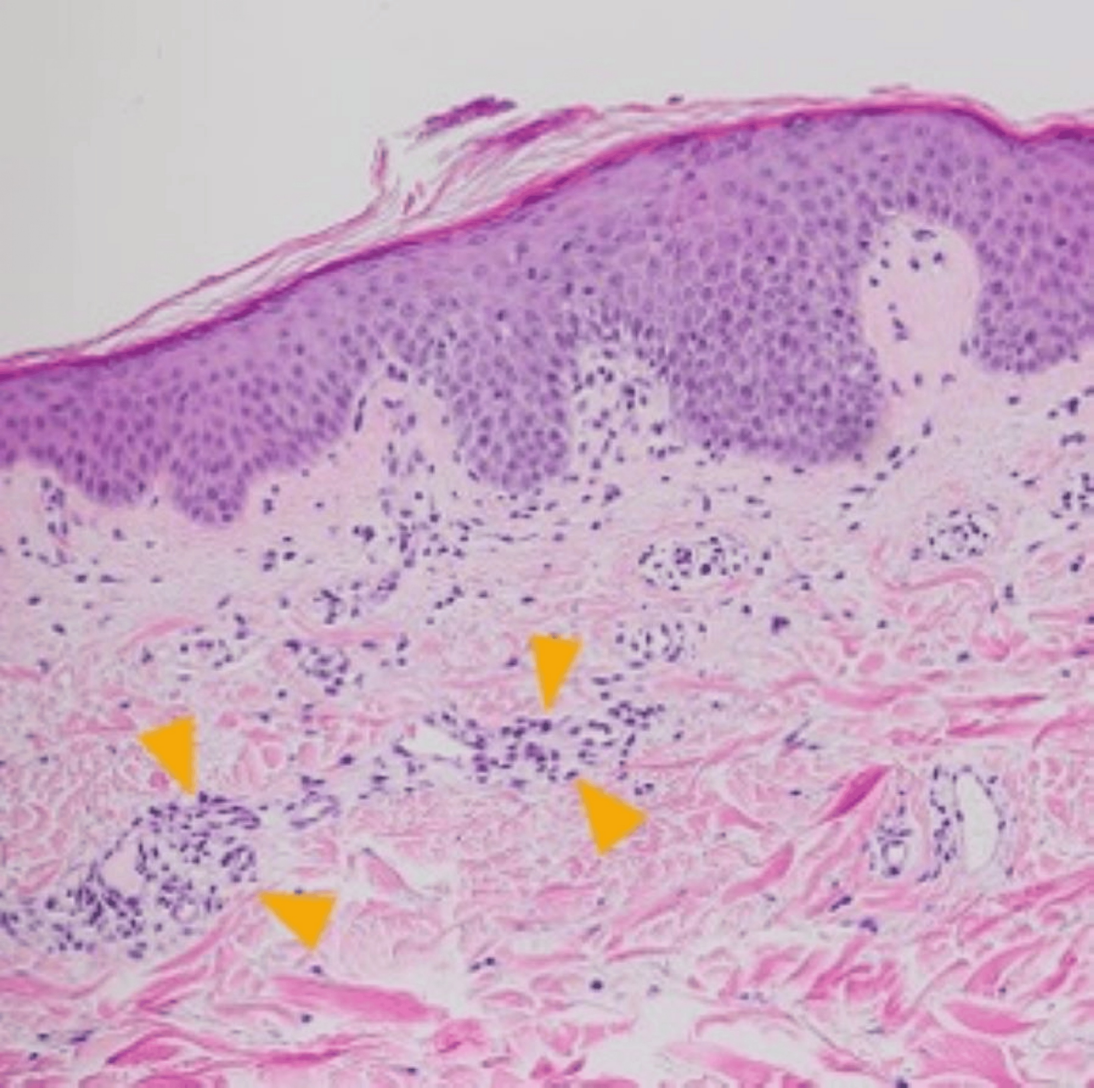
Skin biopsy. A skin biopsy from erythema on the back indicates lymphocyte infiltration around vessels in the upper dermis, known as superficial perivascular dermatitis (arrowhead) (hematoxylin and eosin, ×100).

The rings were single-layered and expanded outward, with slightly raised edges and no signs of infiltration or desquamation. A skin biopsy from the erythematous lesion on his back revealed superficial perivascular dermatitis (Figure 2).

The rash was diagnosed as a superficial type of EAC. Initially, however, it was considered an exacerbation of psoriasis; cyclosporine was prescribed at 2.5 mg/kg per day for 12 weeks, but no improvement was observed. Autoimmune bullous disease or Sjögren's syndrome was also considered as a possible cause of refractory erythema annulare, but testing ruled them out (Table 1).

**Table 1:** Laboratory findings. Alb: albumin; ALT: alanine transaminase; AST: aspartate aminotransferase; BP: bullous pemphigoid; BUN: blood urea nitrogen; Cl: chloride; Cr: creatinine; CRP: C-reactive protein; Dsg: desmoglein; HCT: hematocrit; HGB: hemoglobin; K: potassium; LDH: lactate dehydrogenase; Na: sodium; PLT: platelet; RBC: red blood cell; SS: Sjögren’s syndrome; TP: total protein; WBC: white blood cell

Laboratory investigation	Results	Reference range
WBC count	5,400/μL	3,300-8,600/μL
Neutrophils	63.6%	41.2%-69.7%
Lymphocytes	21.9%	22.1%-46.9%
Monocytes	9.0%	4.1%-9.6%
Eosinophils	4.0%	0.0%-3.5%
Basophils	1.5%	0.0%-1.1%
RBC count	429 × 10^4^/μL	386-492 × 10^4^/μL
HGB	12.1 g/dL	11.6-14.8 g/dL
HCT	36.7%	35.1%-44.4%
PLT	44.1 × 10^4^/μL	15.8-34.8 × 10^4^/μL
TP	6.9 g/dL	6.6-8.1 g/dL
Alb	3.5 g/dL	4.1-5.1 g/dL
AST	12 IU/L	13-30 IU/L
ALT	17 IU/L	7-23 IU/L
LDH	184 IU/L	124-222 IU/L
BUN	17.4 mg/dL	8.0-20.0 mg/dL
Cr	0.80 mg/dL	0.46-0.79 mg/dL
Na	141 mEq/L	138-145 mEq/L
K	4.5 mEq/L	3.6-4.8 mEq/L
Cl	104 mEq/L	101-108 mEq/L
CRP	0.96 mg/dL	0.00-0.14 mg/dL
Anti-BP180 antibody	8.9 U/mL	<9.0 U/mL
Anti-Dsg1 antibody	<3.0 U/mL	<20.0 U/mL
Antinuclear antibody	<40 antibody titer	<40 antibody titer
Anti-SS-A antibody	<1.0 U/mL	<7.0 U/mL
Anti-SS-B antibody	<1.0 U/mL	<7.0 U/mL

**Figure 3:**
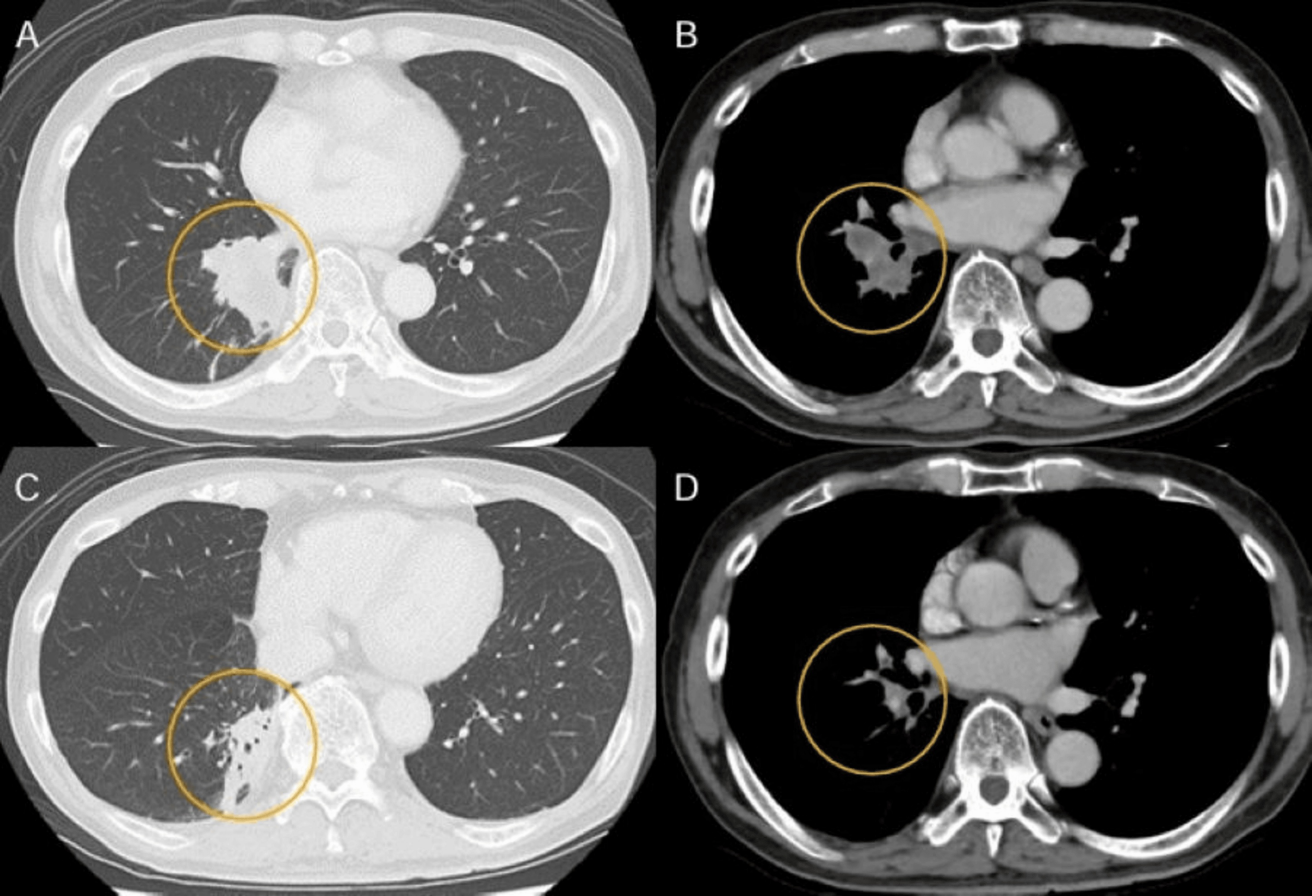
Chest contrast-enhanced computed tomography (CT). CT images reveal a mass in the right lower lobe (A) and swelling in the hilar lymph node (B). After two months of osimertinib treatment, the CT scan indicates shrinkage of the lung cancer (C) and lymph nodes (D).

Infections and drug reactions were also ruled out as potential causes. Paraneoplastic skin syndrome was suspected due to severe pruritus and refractory erythema annulare. A head-to-toe computed tomography scan revealed a mass in the right lower lobe and swelling of the hilar and mediastinal lymph nodes (Figures 3A, 3B).

**Figure 4:**
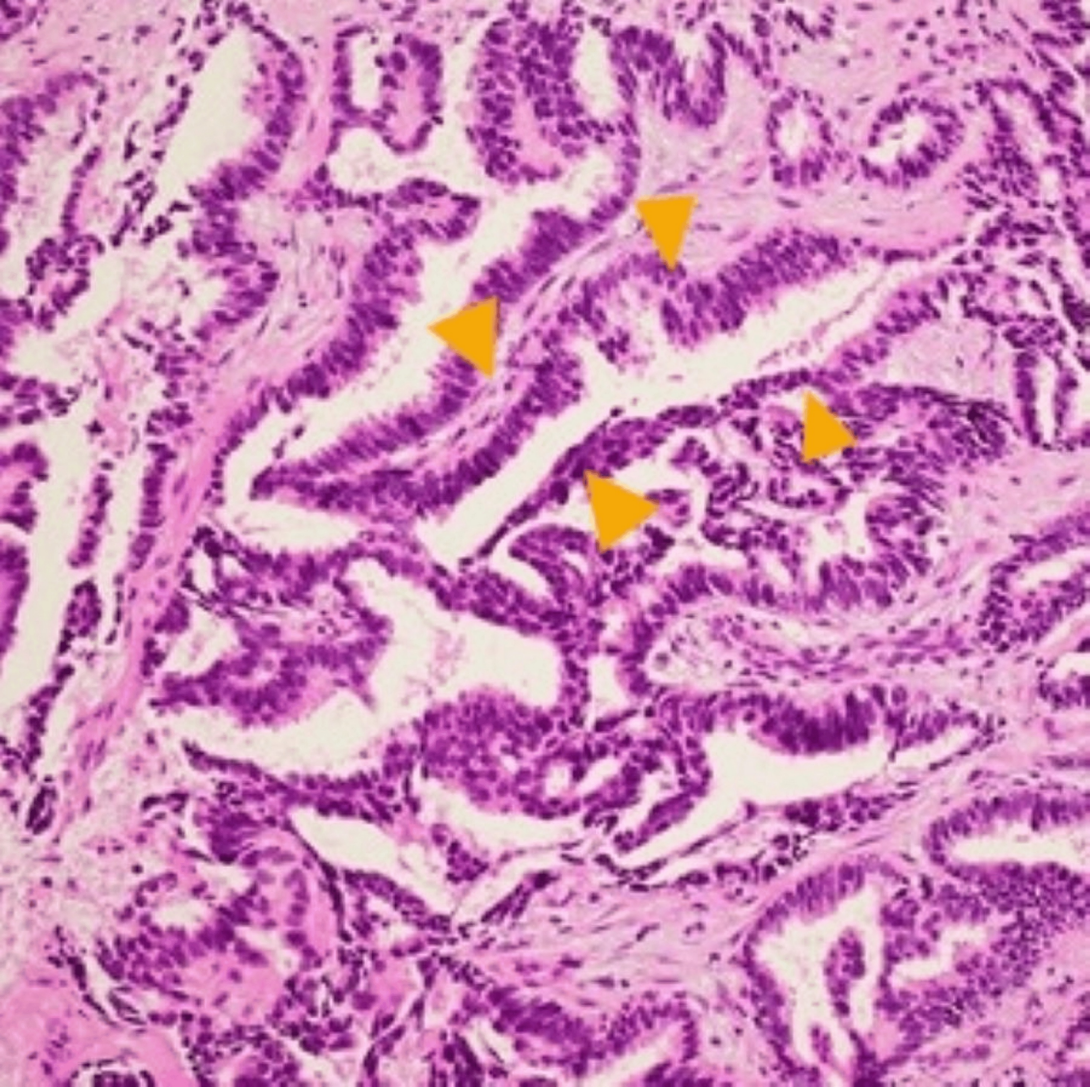
Lung biopsy. A bronchoscopy biopsy reveals irregular tubular and papillary proliferation of atypical columnar epithelium, consistent with adenocarcinoma (arrowhead) (hematoxylin and eosin, ×200).

Four months after the skin rash worsened, the patient was diagnosed with epidermal growth factor receptor mutation-positive lung adenocarcinoma via bronchoscopy (Figure 4).

The cancer was accompanied by bone metastasis (cT3N2M1c, stage IV, exon 19 del). Treatment with osimertinib at a dose of 80 mg/day was initiated, and the erythema nearly disappeared after one month, leaving only pigmentation (Figures 1C, 1D). Two months later, an assessment demonstrated that the lung cancer and lymph nodes had decreased in size (Figures 3C, 3D). Based on the timeline for the resolution of the skin rash during lung cancer drug treatment, the rash was ultimately diagnosed as PEACE associated with lung cancer.

## Discussion

The clinical course of this patient highlights two significant issues. First, EAC may resolve with drug therapy for lung cancer. Second, a thorough investigation for suspected lung cancer is warranted when EAC is present.

The observation that erythema annulare disappeared within one month of initiating osimertinib suggests that targeted therapy for lung cancer can lead to the resolution of the skin rash. Except for our case, only one case has been reported where lung cancer drug treatment improved EAC [7]. In that case, the histological analysis indicated squamous cell lung carcinoma, and the patient was treated with gemcitabine and carboplatin. It is suggested that the pathogenesis of EAC possibly occurs through T helper 1 cell-mediated reactions and tumor necrosis factor-alpha, as well as proinflammatory cytokines [7-8]. In our case, the lung cancer drug therapy may have consequently suppressed the reaction. To the best of our knowledge, our case is the first case in which the EAC disappeared after treatment with osimertinib.

The patient was initially diagnosed with psoriasis, a condition generally not associated with EAC [8]. Therefore, we cannot dismiss the possibility that the skin rash may have been due to PEACE from the outset, particularly given the patient's history of ascending colon cancer.

For the second clinical issue, a systematic evaluation for potential lung cancer is recommended when EAC is present. While EAC has predominantly been linked to leukemia and lymphoma in cases of internal malignancies [3], its association with lung cancer, although rare, warrants attention due to the generally poor prognosis associated with advanced lung cancer.

## Conclusions

EAC may resolve with targeted drug therapy for lung cancer, suggesting that a thorough evaluation for suspected lung cancer is warranted when EAC is present. It is intriguing to consider that changes in skin rashes may predict the efficacy of lung cancer treatment or indicate recurrence risk. However, it is necessary to gather more cases, as "hidden" lung cancer or "hidden" PEACE may be more prevalent than we currently realize.
